# Gene Amplification and the Extrachromosomal Circular DNA

**DOI:** 10.3390/genes12101533

**Published:** 2021-09-28

**Authors:** Noriaki Shimizu

**Affiliations:** Graduate School of Integrated Sciences for Life, Hiroshima University, 1-7-1 Kagamiyama, Higashi-Hiroshima 739-8521, Hiroshima, Japan; shimizu@hiroshima-u.ac.jp

**Keywords:** gene amplification, extrachromosomal DNA, double minutes, micronucleus, cancer, genome plasticity, chromothripsis, gene expression, repeat-induced gene silencing

## Abstract

Oncogene amplification is closely linked to the pathogenesis of a broad spectrum of human malignant tumors. The amplified genes localize either to the extrachromosomal circular DNA, which has been referred to as cytogenetically visible double minutes (DMs), or submicroscopic episome, or to the chromosomal homogeneously staining region (HSR). The extrachromosomal circle from a chromosome arm can initiate gene amplification, resulting in the formation of DMs or HSR, if it had a sequence element required for replication initiation (the replication initiation region/matrix attachment region; the IR/MAR), under a genetic background that permits gene amplification. In this article, the nature, intracellular behavior, generation, and contribution to cancer genome plasticity of such extrachromosomal circles are summarized and discussed by reviewing recent articles on these topics. Such studies are critical in the understanding and treating human cancer, and also for the production of recombinant proteins such as biopharmaceuticals by increasing the recombinant genes in the cells.

## 1. Gene Amplification and the Extrachromosomal Circles in Human Cancer

The amplification of oncogenes or drug-resistant genes plays a pivotal role in human cell malignant transformation by conferring growth advantage to the cells through the overproduction of the amplified gene product. A classical cytogenetic study located the amplified genes at the extrachromosomal double minutes (DMs) or the chromosomal homogeneously staining region (HSR) [1]. DMs and HSR mutually interconvert [2,3], and share the same sequence [4]. DMs are stable extrachromosomal elements that contain circular DNA. Circularity has been suggested based on electron microscopy [5], sensitivity to radiation-mediated breakage [6], and the absence of telomeric structures [7]; this was recently re-enforced by integrating ultrastructural imaging, long-range optical mapping, and computational analysis of whole-genome sequencing [8]. In contrast, cytogenetically undetectable circular DNA has been identified in many normal and cancer cell lines and normal tissues more than three decades ago [9]. Recently, many reports have described circular extrachromosomal DNA in normal or cancer cells [10]. In general, the circles in normal cells [11,12] were smaller in size (less than 1 kbp) than those in cancer cells (1–2 Mbp) [13]. The former is referred to as extrachromosomal closed circular DNA (eccDNA), and the latter are referred to as extrachromosomal DNA (ecDNA). EcDNAs are equivalent to conventional DMs; however, the term ecDNA was recently used instead of DMs because it does not always appear as a doublet among the chromosome spread specimens. Several extensive studies that used a large number of clinical samples together with the most advanced techniques, unambiguously, reinforced the tight relationship between malignancy and the appearance of ecDNA/DMs [13,14].

It is important to note that gene expression from the same amplicon sequence is higher in the extrachromosomal context than in the chromosomal context [15] because the chromatin of extrachromosomal DNA is more favorable for gene expression [8,16]. Consistently, DMs were replicated early in the S phase, while the HSRs of the same amplicon were replicated at the end of the S phase [4]. The higher gene expression may reflect the circular nature that poses a topological constraint that favors DNA helix unwinding [8]. Alternatively, I now propose that it may reflect the plausible localization of extrachromosomal elements in the interchromosome domain (ICD)compartment, where gene expression is favored [17].

## 2. Intra-Cellular Behavior of the Extrachromosomal Circles

As described above, oncogene amplification contributes to the malignancy of human cells. Conversely, the elimination of amplified genes from cancer cells results in cellular differentiation, growth arrest, and apoptotic cell death [18,19,20]. Therefore, if we could eliminate the DMs/ecDNA-bearing amplified oncogenes, we could cure many types of cancers. The extrachromosomal DMs are devoid of both telomeres [7] and centromeres [1]/kinetochores [21]. Therefore, the number of such acentric elements per cell fluctuates during cell proliferation. Such fluctuations may generate genetic heterogeneity among cells in the cancer tissue [22]. Furthermore, targeted therapy resistance develops if the amplified genes are localized at the extrachromosomal circles [23].

**Figure 1:**
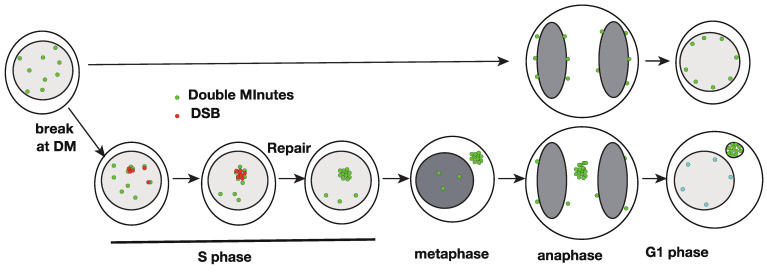
A model explaining how double minute (DM) breakage results in their aggregation, repair, and micronucleation. DM-derived sequences are shown in green, double-strand breakage (DSB) is shown in magenta, and chromatin is shown in gray. Modified from Figure 6G in [30].

The acentric DMs should stick to the mitotic chromosome arm during mitosis and cytokinesis to segregate to the daughter cell nucleus [24], similar to the strategy used by the nuclear episomes of many DNA viruses (reviewed in [25]). Detachment from the chromosome arm results in cytoplasmic localization after mitosis [26]. On the other hand, low concentrations of replication inhibitors such as hydroxyurea (HU) induced the cytoplasmic micronuclei that were highly enriched with DMs [18,27]. The same conditions also induced the elimination of DMs bearing the amplified genes [18,19,20]. Therefore, the elimination of DMs might be mediated by entrapment into the cytoplasmic micronucleus. The incorporation was highly selective; thus, purification of such micronuclei provided almost pure DM DNA [28]. Subsequent studies revealed that such micronuclei were derived from the intra-nuclear aggregates of numerous DMs (see Figure 1), namely, a low concentration of HU induced double-strand breakage throughout the nucleus, and HU also induced aggregation of numerous DMs in the nucleus [29]. The CRISPR/Cas9-induced specific breakage of DMs was sufficient for aggregation and subsequent micronucleation of DMs [30]. Homologous recombination machinery may be involved in the aggregation process because it occurs only after the S phase. Such aggregates of DMs did not stick to the chromosome, were left behind the separating anaphase chromosomes, and generated micronuclei with almost pure DMs [26,31].

Interestingly, the linear DNA microinjected into the nucleus rapidly aggregated [32], suggesting that numerous damaged DNA, in general, were aggregated. Such aggregated DNA could pass through the interphase nuclear membrane and appear in the cytoplasm of living cells [32]. Similarly, nuclear budding or nuclear herniation (rupture) [33,34] generated cytoplasmic chromatin. Nuclear budding was induced by a large cytoplasmic bleb (protrusion), which was induced by fresh serum or the microtubule inhibitor nocodazole [31]. Such cytoplasmic blebs pulled out the chromatin from the interphase nucleus through the lamina break. This process generates cytoplasmic micronuclei without lamina [31]. This has important implications because chromatin in the cytoplasm stimulates the cGAS-STING pathway, which evokes an inflammatory response (reviewed in [35]).

## 3. Generation of DMs/EcDNA and HSR from Small eccDNA

**Figure 2:**
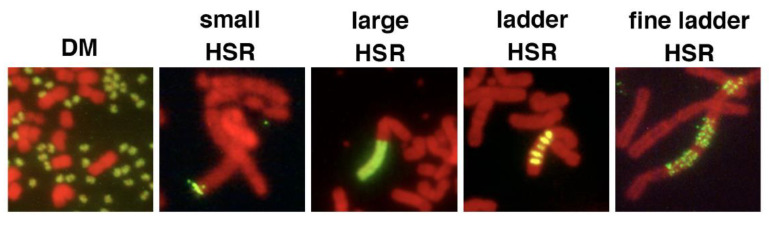
Amplification of IR/MAR-bearing circular plasmid in the transfected cells. An IR/MAR plasmid was transfected to human colorectal carcinoma COLO 320DM cells. Stable transformants were selected by drug for more than one month. The chromosome spread was hybridized with a probe prepared from the transfected plasmid. The hybridized probe was detected by green fluorescence, and the DNA was counterstained with propidium iodide (shown in red). The plasmid generated extrachromosomal double minutes (DM) or several types of homogeneously staining region (HSR). The photos of DM [43] and large HSR [38] appeared previously, and the other photos are unpublished.

**Figure 3:**
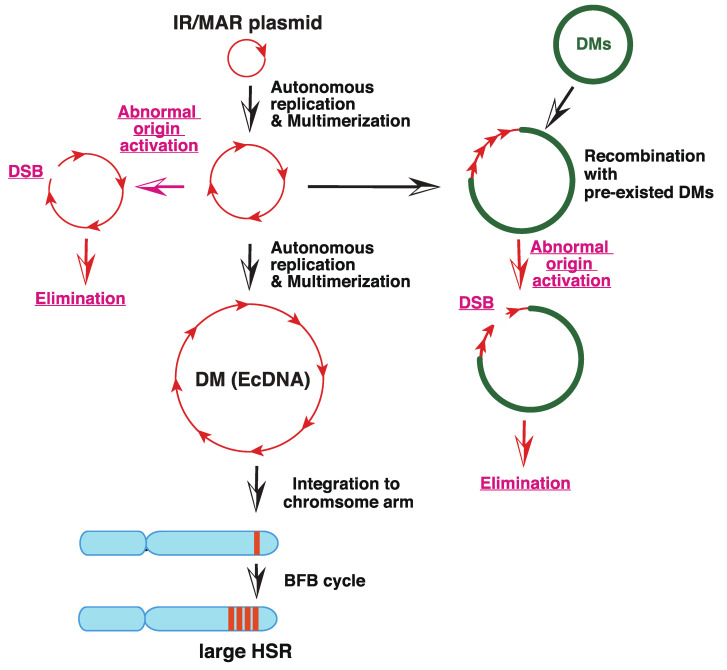
Mechanism of amplification of an IR/MAR-bearing circular plasmid. The IR/MAR plasmid is shown in red arrows. DMs, if pre-existed in the same cells, are shown in green, and the chromosome arm is shown in cyan. The processes in wild-type cells are indicated by black arrows, and the ones only in *SIRT 1* knock-out cells are indicated by red arrows. Modified from Figure 7 of [44].

“The episome model” of gene amplification [2,36] argued that the submicroscopic circular episome derived from the chromosome arm was maintained and multimerized to generate larger DMs. If such a circle is integrated into the chromosome arm, it induces the breakage-fusion-bridge cycle (BFB) and generates chromosomal HSR. This hypothesis was demonstrated using a plasmid bearing a replication initiation region (IR) and a nuclear matrix (scaffold) attachment region (MAR/SAR), both of which are required for replication initiation. Such plasmids, if transfected into human colorectal carcinoma COLO 320DM cells, spontaneously and efficiently generated DMs and/or HSRs in stable transformants, which were morphologically indistinguishable from those detected in malignant cells [37,38]; see Figure 2). For amplification, both IR and MAR were required. The minimum sequence required for efficient amplification was isolated from *DHFR*, c-*myc* [39], and β-*globin* IR [40], and such core IR has many kinds of sequence elements that are required for replication initiation. The mechanism of gene amplification was studied using this system (Figure 3, black arrows). The circular plasmid DNA with IR/MAR was multimerized to large circles, where the sequences were arranged in tandem repeats [38]. The large circle may be identified as DMs under light microscopy if it becomes sufficiently large [41]. The tandem repeat of the IR/MAR plasmid was then integrated into the chromosome arm, where it efficiently initiated the BFB cycle that generated HSR [41,42].

Importantly, the IR/MAR sequences that support gene amplification are scattered throughout the human genome because replication is initiated at ca. 100 kbp intervals [45]. Therefore, among the numerous small eccDNAs generated from the chromosome arm, at least a portion of them should be amplified similarly to the IR/MAR plasmid. Furthermore, any DNA co-transfected with the IR/MAR plasmid was efficiently co-amplified in the transfected cells [38], suggesting frequent recombination between the extrachromosomal DNA. This was consistent with the fact that natural DMs/ecDNA were a patchwork of sequences derived from several separate chromosome regions [46,47]. Such co-amplification of extrachromosomal circles drives the co-amplification of distantly located enhancer sequences together with the oncogene, thus enhancing the expression of oncogenes [48]. Furthermore, the efficiency of IR/MAR gene amplification varied significantly between normal and tumor cells as well as between the different tumor cell lines ([43,49] our unpublished data). This may correspond to the fact that DM/ecDNA and/or gene amplification is restricted to certain types of cancer cells [13], and may reflect that the stability of the circles bearing the IR/MAR sequence is only limited to amplification-prone cell types [49]. We do not know which gene may determine the amplification phenotype; however, SIRT1 stabilizes the extrachromosomal element [44]; Figure 3, red arrows by preventing activation of latent origin of replication initiation [50].

## 4. From Chromosome Arm to Gene Amplification

The episome/eccDNA bearing the IR/MAR sequence was multimerized to generate larger and complex DMs/ecDNAs. The mechanism that generates an initial small circle from the chromosome arm was discussed as follows: The most plausible mechanism is chromothripsis, which is mediated by micronuclei. Chromothripsis has been suggested by cancer genomics, and it involves the abrupt fragmentation of a specific chromosome followed by re-ligation and extensive rearrangement of many fragments [51,52]. The fragmentation of a specific chromosome might occur in micronuclei [53,54] if the nuclear membrane of the micronuclei ruptures [55,56]. It has been reported that replication [57] and transcription [58] are defective in lamina-negative micronuclei. The re-ligation of the fragment produces a large number of circular molecules [59]. Among such circles, the circles with IR/MAR would be amplified as described above. A model system reproduces this process in culture [49]. It is known that human chromosomes are specifically eliminated in human-rodent hybrid cells. In such hybrids, the human chromosome was selectively incorporated into micronuclei because of the malfunctioning of the human centromere in such hybrids. Then, the micronuclear content was broken, and the human chromosome was eliminated. Importantly, there remained numerous acentric stable DMs with a mark of the human genome, that is, Alu, among stable rodent chromosomes. Such DMs are composed of a patchwork of sequences derived from multiple human chromosome regions, consistent with the structure of natural DMs/ecDNA in human cancer [46].

## 5. Applications of the Extrachromosomal Element-Mediated Gene Amplification

The circular plasmid DNA bearing the IR/MAR mimics gene amplification, thus providing an excellent model to study genetic plasticity associated with human malignancy. Furthermore, the system provides a novel platform for recombinant protein production, whose efficiency needs to be increased, especially in the case of biopharmaceutical production. However, this application has two major limitations. One is the cell-type dependency of the amplification efficiency ([43] our unpublished results). The problem was technically solved by amplifying the target genes on the artificial chromosome [60,61] in the amplification-prone cells, followed by its transfer to the amplification-difficult cells by micronuclei-mediated chromosome transfer [61]. Another problem was that the amplification produced an ordered tandem repeat, which was subjected to repeat-induced gene silencing (RIGS; [62,63]). RIGS is an important cellular mechanism that heterochromatinizes the pericentric region to increase mechanical strength [64], prevent transposon spreading [65], or silence transgenes [66,67]. The problem was, at least in part, overcome by the finding that RIGS is sequence-dependent [68]. Some sequences, which included the core IR [69], the MAR, or the human genomic B-3-31 sequence, resulted in a reverse phenomenon, that is, repeat-induced gene activation (RIGA), while other sequences, which included bacterial plasmid, phage, or human transposon sequences, resulted in RIGS. Furthermore, knock-out of a histone deacetylase SIRT1 might alleviate RIGS, in combination with butyrate treatment, which inhibits another type of histone deacetylase [44]. Therefore, we are now able to amplify sequences of interest that are not subject to RIGS. We anticipate an increase in recombinant production in a gene number-dependent manner from the amplified recombinant genes.

## 6. Future Task

There is no doubt about the importance of circular extrachromosomal DNA for cancer development. Much has been understood about what they are, how they are generated, and how they behave in cells. However, the following questions need to be addressed. (1) How were the small eccDNAs generated from the chromosome arm? The detailed molecular mechanisms should be clarified. (2) Which portion of eccDNA is stable and contributes to gene amplification? Such a stable circle should contain at least the IR/MAR sequence, which is required for extrachromosomal replication, multimerization, and recombination with other circles. Furthermore, some additional sequence(s) may be required for the stable segregation of daughter cells by sticking to the mitotic chromosome. (3) What genetic background of the cells supports the stability of the circle? It would likely determine the amplification-prone phenotype of certain tumor cells and would be crucial for cancer diagnostics. This understanding is important for industrial applications.

**Figure 4:**
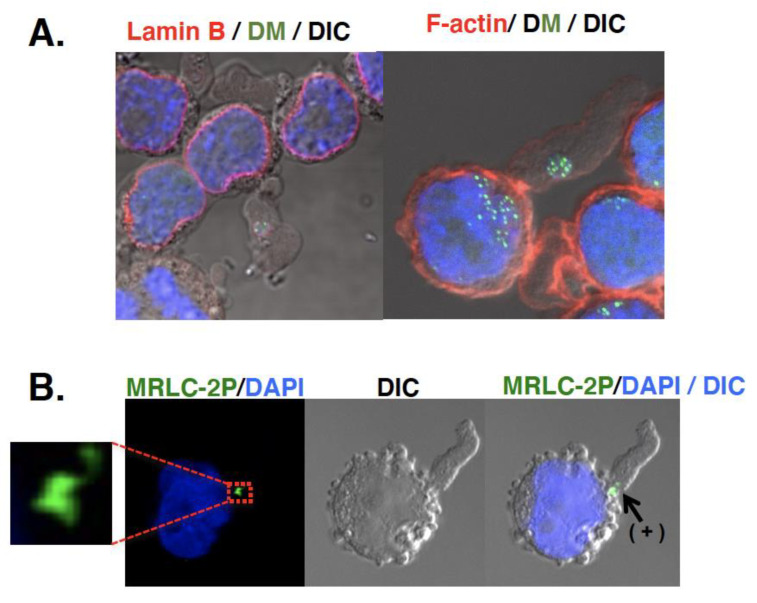
Possible involvement of cytoplasmic bleb in the elimination of extrachromosomal circles. Large cytoplasmic bleb (protrusion) induced by fresh serum could entrap the micronuclei [31]. The bottom of such bleb was constricted (grayscale DIC image), where actin (red in (**A**)) and phosphorylated myosin (MRLC-2P; green in (**B**)) was located (noted as “+” in (**B**)) just like a contractile ring during the cytokinesis. DMs were detected in green by Lactose repressor-GFP binding to lactose operator sequence on DMs [29]. These are unpublished images.

Another important question is the fate of the micronuclei. As described, the initial study [20,28] suggested the involvement of micronuclei in the elimination of DMs/ecDNA. A later study uncovered the mechanism by which DMs/ecDNA are selectively entrapped by micronuclei [26,29,30]. However, the question of how the micronuclei content is eliminated has not yet been clarified. Micronuclei were detected in culture fluid [70]. Such extracellular micronuclei were enriched with DMs/ecDNA, had intact lamina, non-damaged DNA, and cytoplasmic membrane. Large cytoplasmic blebs, which were induced by the addition of fresh serum, might entrap the micronuclei [31]. The bottom of the bleb was constricted, where actin and phosphorylated myosin were located just like a contractile ring during cytokinesis (Figure 4). Therefore, such blebs were easily broken by fluid flow, releasing the extracellular micronuclei (unpublished observation). Consistent with this, microvesicles with amplified oncogene DNA [71] or ecDNA [72] were detected in human plasma, which is useful for cancer diagnostics. Such a process is important and ought to be addressed.

Clarifying these questions is important in understanding and treating human cancer as well as for industrial applications.

## Data Availability

Not applicable.
